# Relation between Blood Pressure Variability within a Single Visit and Stroke

**DOI:** 10.1155/2021/2920140

**Published:** 2021-03-02

**Authors:** Wei Ma, Ying Yang, Litong Qi, Baowei Zhang, Lei Meng, Yan Zhang, Min Li, Yong Huo

**Affiliations:** Division of Cardiology, Peking University First Hospital, Dahongluochang Street, Xicheng District, Beijing 100034, China

## Abstract

Blood pressure variability (BPV) has been identified as an important risk factor for cardiovascular events. The white coat effect (WCE), which is measured as the first systolic blood pressure (SBP) measurement minus the mean of the second and third measurements, is a BPV indicator within a single visit. In total, 2,972 participants who had three measurements of BP within a single visit were included. The participants were divided into three groups based on their WCE percentiles: Group 1 (WCE_2.5-97.5_, 2.5–97.5th percentiles of WCE), Group 2 (WCE_2.5_, 0–2.4th percentiles of WCE), and Group 3 (WCE_97.5_, 97.6–100th percentiles of WCE). A multiple logistic regression model was used to analyze the relationship between WCE and stroke after adjusting for cardiovascular disease risk factors. Compared with the WCE_2.5-97.5_ group, the OR for stroke in the WCE_2.5_ group was 2.78 (95% CI: 1.22, 6.36, *p*=0.015). After adjusting for cardiovascular factors, OR increased to 3.12 (95% CI: 1.22, 7.96, *p*=0.017). The OR of WCE for stroke was 0.93 (95%CI: 0.87, 0.99, *p*=0.036). BPV within a single visit is associated with stroke. The value and direction of the change may be important as well.

## 1. Introduction

Blood pressure variability (BPV) is increasingly being recognized as another essential parameter in risk prediction for cardiovascular events and mortality [1]. Long-term BPV is associated with stroke [2]. In addition, visit-to-visit SBP variability is an independent predictor of primary stroke in Chinese hypertensive patients [3]. Home day-to-day BPV is also associated with an increased risk of stroke [4]. Our previous study showed that carotid atherosclerosis can predict ischemic cardiovascular disease events including stroke [5]. There are various methods to evaluate BPV; however, clinically validated protocols and criteria are still lacking. Short-term BPV, as assessed by 24-hour ambulatory blood pressure monitoring, was first assessed in the pioneering paper by Parati et al. [6], while the variability of BP measured on different clinical visits has been used to evaluate long-term BPV by Rothwell et al. [7] cases highlight the role of an increased BPV as a prognostic marker.

The phenomenon of BPV within a single clinical visit has also been recognized for a long time [8]. Two blood pressure readings should be taken on each visit, per the 2013 ESH/ESC hypertension guidelines [9], and if the readings vary by > 5 mmHg, additional readings should be taken until the two are close [10]. However, the selection of 5 mmHg as a cutoff point for additional blood pressure measurements is arbitrary. Less is known concerning the patterns of within-visit BPV. Recent studies have demonstrated that within-visit BPV is associated with cardiovascular risk factors [11] and the risk of stroke [2] but not with overall cardiovascular disease or all-course mortality [12].

How to evaluate BPV within visit still needs to be clarified. SD, CV, and the range of the three BP measurements within a single visit were used. The white coat effect (WCE) within a single visit was adopted in a foundational study [7] as the first SBP measurement minus the mean of the second and third SBP measurements, and it is one type of within-visit BPV. Hypertensive patients treated mainly with amlodipine compared with those treated mainly with atenolol had a lower WCE [7]. The WCE can reflect not only the value but also the direction of the BPV within a single visit. However, little data are available on the prognostic importance of BPV obtained during a single clinic visit. To address this issue, we investigated the WCE value within a single visit as a way to monitor stroke risk in a Chinese community population. If it is associated with a specific CVD risk, within-visit BPV could be a clinically useful measure because it can be assessed in a single visit.

## 2. Methods

### 2.1. Population

The current study included adult subjects registered in two local community hospitals in an urban district of Beijing, China. In the Pingguoyuan community, the survey was conducted from September to December 2007. The community had 42,500 (aged 18 years or above) residents. A subgroup within this community was selected by cluster sampling, and then a proportion sampling method was used for final selection. Finally, 1,497 people were recruited. The survey in the Gucheng community was conducted from April to September 2008. Residents were contacted by telephone or by recruitment advertisements, and those volunteering to participate were included. A total of 1,531 participants from this district were recruited, and they were all aged 40 years or older. The characteristics of this group have been detailed in a previous study [13]. In total, 3,028 participants from these two communities were recruited, among which analyses were carried out on 2,972 (98.15%) individuals who had three BP measurements within a single visit. The study was approved by the institutional review board of Peking University First Hospital, and informed consent was obtained from all participants.

### 2.2. Measurement of Cardiovascular Risk Factors and Stroke

Body mass index (BMI) was calculated using height and weight measurements. After a rest period of 15 minutes, BP was measured three times with at least 2 min intervals between each measurement; a nurse took these measurements on the patient's right upper arm while in a sitting position and by using a mercury sphygmomanometer in the morning. The mean systolic BP (SBP) and diastolic BP (DBP) were calculated based on the three measurements. Heart rate (HR) was obtained during the first BP measurement. A fasting blood sample was collected for the analysis of total cholesterol (TC), triglycerides (TG), and serum creatinine using standard techniques in the Beijing Hypertension League Institute. Estimate glomerular filtration rate (eGFR) was calculated according to CKD-EPI formula [14]. The eGFR <60 mL/min/1.73 m^2^ was defined as chronic kidney disease (CKD) [15]. Participants with a history of cigarette smoking were identified as smokers. Hypertension was defined as office SBP ≥140 mm Hg and/or DBP ≥90 mm Hg or history of hypertension. The usage of antihypertensive drugs was reviewed as well. Diabetes was diagnosed according to the patient's interview. Myocardial infarction (MI) was defined by a history of acute MI, and if pathological Q waves or coronary T waves were noted in the electrocardiogram (ECG) and correspondingly regional wall motion abnormality was confirmed by echocardiography simultaneously [16]. Stroke, including cerebral infarction, intracerebral hemorrhage, and transient ischemic attack, was determined based on the history of data collected from hospitalizations and outpatient records, which were confirmed by CT  or MRI scan [16].

### 2.3. Carotid Artery Ultrasound and ba-PWV Measurement

Carotid ultrasonography was conducted by the General Electric vivid I apparatus, which was equipped with a high-resolution 10 MHz linear array transducer. Optimal longitudinal and transverse B-scan images were obtained and stored on a compact disc. The data were measured by two experienced ultrasonologists in the Central Laboratory of Echocardiography of Peking University First Hospital. The examination and measurement methodology followed a protocol previously described [13, 16]. Three measurements were obtained for each site at 5 mm intervals at the end of the cardiac diastole. For each individual, carotid IMT (cIMT) was determined as the average of the IMT values in 36 sites, including three points at the anterior and posterior wall of the common carotid artery, carotid bifurcation, and internal carotid artery of both sides. Plaques were avoided when taking cIMT measures [17]. CIMT thickening was defined as cIMT ≥ 0.9 mm [18]. Carotid plaque was defined as a focal part protruding into the lumen with a maxIMT ≥ 1.3 mm or a focal raised lesion >0.5 mm with or without flow disturbance [19]. The reproducibility of carotid IMT measurements of these two groups according to this protocol was assessed and found to be acceptable. A better reproducibility was found when measuring the mean IMT rather than the max IMT when focusing on CCA and Bulb IMT rather than on ICA IMT and when targeting the far wall IMT rather than near wall IMT [20].

Brachial-ankle pulse wave velocity (ba-PWV) was measured using a VP1000 vascular profiler (Omron Colin, Japan) after at least 5 min rest. Details of the measurement have been described in a previous report [21]. Left and right side ba-PWV were measured at the same time, and the higher value of ba-PWV was considered for data analysis. Ba-PWV ≥ 1400 cm/s was defined as ba-PWV abnormality [22].

### 2.4. Statistical Analysis

The WCE within a single visit was assessed as the difference between the first SBP measurement and the mean of the second and third measurements [7]. Because there is no reference value for identifying a “normal” value, we classified the magnitude of the WCE as a function of its percentile distribution. The participants were divided into three groups according to the degree of their WCE: Group 1 (WCE_2.5-97.5_, including WCE values within the 2.5–97.5th percentiles of WCE, with −4 mmHg ≤ WCE ≤ 6 mmHg), Group 2 (WCE_2.5_, 0–2.4th percentiles of WCE, with WCE < −4 mmHg), and Group 3 (WCE_97.5_, 97.6–100th percentiles of WCE, with WCE>6 mmHg). According to the Kolmogorov–Smirnov test, the distribution of continuous variables, such as age, BMI, SBP, DBP, WCE, HR, cIMT, TC, TG, and ba-PWV, was skewed among the three groups. Therefore, those variables were expressed as medians plus quartiles, and the Kruskal–Wallis H test was used to compare them among the three groups. Category variables were presented as percentages, and a Pearson's *χ*^2^ test was used to compare them among the three groups.

A univariate logistic regression model was used to analyze the association of various traditional cardiovascular risk factors (age, BMI, gender, SBP, DBP, diabetes, hypertension, TC, TG, smoking status, etc.), degree of WCE and WCE groups, cIMT thickening, carotid plaque, PWV abnormality, and stroke. Group 1 of WCE was used as reference. A multiple logistic regression model was used to further analyze the relation between WCE and stroke by adjusting for age, BMI, gender, smoking status, TC, MI, CKD, SBP, hypertension, antihypertensive drug usage, diabetes mellitus, IMT thickening, ba-PWV abnormality, and carotid plaque. Subgroup analyses and interaction tests were conducted to examine the relationships between WCE and stroke according to age group (<65 years and ≥65 years), sex (male and female), smoking (yes or no), hypertension (yes or no), diabetes mellitus (yes or no), carotid plaque (yes or no), carotid IMT thickening (yes or no), and PWV abnormality (yes or no).

A *P* value < 0.05 was considered statistically significant. All analyses were performed using SPSS software (version 14.0, SPSS).

## 3. Results

The distribution of the WCE is shown in Table 1. The WCE in the WCE_2.5_ group was less than −4 mmHg, which showed that in some participants, SBP1 was less than the other two measurements. The general characteristics of the three WCE groups are shown in Table 2. There was no difference in the traditional risk factors for stroke among the three groups for WCE, except for SBP, WCE, and carotid plaque percentiles.

The univariate analysis showed that traditional risk factors, such as age, gender, hypertension, diabetes, obesity, SBP, and hypercholesterolemia, were related to stroke (*P* < 0.05). Other new noninvasive indicators of atherosclerosis, such as cIMT thickening, carotid plaque, and ba-PWV abnormality, were also related to stroke (*P* < 0.05) (Table 3). The OR for stroke was 2.78 (95% CI: 1.22, 6.36, *P*=0.015) in the WCE_2.5_ group compared with the WCE_2.5-97.5_ group (Table 3). After adjusting for the above factors, the correlation became stronger; the OR increased to 3.12 (95% CI: 1.22, 7.96, *P*=0.017) (Table 4). The WCE for OR was 0.93 (95%CI: 0.87, 0.99, *P*=0.036) as well. When another category method was adopted, in the WCE_5_ group, the OR for stroke was 1.71 (95% CI: 0.94, 3.11, *P*=0.077) compared with the WCE_5-95_ group (Table 3). This difference did not achieve statistical significance despite the clear trend observed.

**Figure 1:**
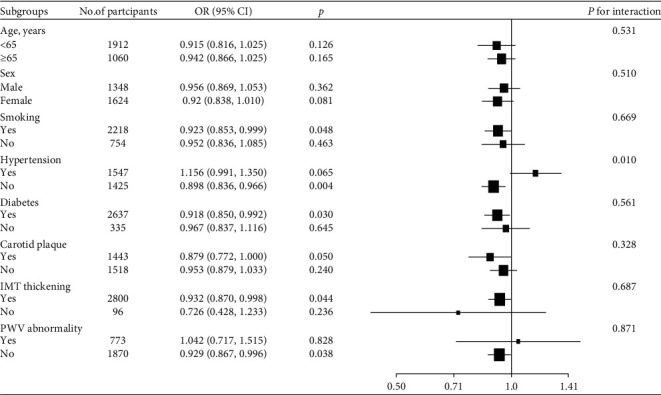
Subgroup analysis of WCE and stroke.

Subgroup analysis is shown in Figure 1. Among the participants who had no hypertension, WCE was negatively correlated with stroke. There was no difference in different age group, sex, smoking status, with or without diabetes, carotid plaque, IMT thickening, and PWV abnormality.

## 4. Discussion

Short-term and long-term BPV has been recognized for a long time [23]. Clinical studies have shown that increased long-term BPV is associated with cardiovascular disease [24], especially stroke [2, 3]. Our study showed that short-term BPV, even BPV within a single visit, may correlate to stroke. Subgroup analysis showed that in the participants who had no hypertension, WCE was negatively correlated with stroke as well.

Different parameters can be used to evaluate BPV within a single visit, such as SD, variable coefficient range, and alarm reaction (defined as the first BP measurement minus the second and/or the third BP measurement) [7, 11, 12]. Systolic BP could rise as high as 74 mmHg within a single visit [25]. BPV within one visit is not rare, and among the three measurements within one visit, the prevalence of the difference between the maximum and minimum SBP being more than 10 mmHg was 19.9% [26]. The within-visit BPV obtained during a single visit and its reflection of a transient fluctuation of BP has been applied more often to evaluate variations in emotional and sympathetic activity [26]. For most people, the first measurement among the three measurements is higher than the other two, so most WCE measurements are positive [11]. A decreasing trend (BP_1_ > BP_2_ > BP_3_) was observed among three consecutive measurements of SBP (17%), while the prevalence of increasing trend (BP_3_ > BP_2_ > BP_1_) was 7.4% [27]. Another study in children and adolescents showed from the first to second measurements, SBP decreased in 58% of the patients, did not change in 10%, and increased in 32% [28]. Additionally, it is important to keep in mind that BP will not drop with repeated measurements for a sizable proportion of the population. WCE_2.5_ was negative in the current study and implied the first SBP was lower than the other two measurements. The WCE used in the present study reflected not only the value but also the direction of BPV within a single visit, which may play a more important role in stroke. WCE ≥ 0 and WCE < 0 have been used as variables which indicate the trend of increase vs. decrease (between the 1st vs. 2/3 BP measurement) of the three measurements; the result showed that there was no correlation with stroke. The appropriate cutoff value of WCE should be studied further. We chose the parameters taken over a few minutes apart as the simplest and most clinically translatable measure.

Why we choose WCE_2.5-97.5_ as the reference was another concern of the current study. The available studies did not provide the normal value of the WCE within a single visit. Nevertheless, a similar classification was used in the previous studies. 15 mm Hg was a cutoff point for pathologically increased daytime systolic BPV because this value exceeds the upper 95% CI (14.9 mm Hg) of the average daytime systolic BPV in all 286 patients [29]. We assumed the group that had WCE values ranged from 2.5 to 97.5 percentiles as normal, just like how we defined the medical reference range as usual. WCE as a continuous variable was also negatively independently associated with stroke. In participants without hypertension, the results were similar, which might show that hypertension could play a more important role in the stroke. The first BP that was measured was generally thought to account for the WCE within a visit and was usually dismissed as an unreliable estimation of the casual BP [24]. Ohkubo et al. have also reported that the initial first home BP values were more significantly related to stroke risk than conventional BP values (mean of the two measurements) [30].

BPV within a single visit being associated with stroke was reported in a reanalysis of the ASCOT-BPLA study [7]. Studies have shown that different BPV values within one visit were related with the progression of atherosclerosis and might be the reason for the connection between varying BPV values within one visit and stroke. Masugata et al. reported that variability in SBP (differences between the SBPs of the two measurements within a single visit) within a single clinic visit showed better correlations with arterial stiffness and risk factors for atherosclerosis than the mean SBP. Large SBP variability during a single clinic visit may reflect the progression of atherosclerosis in treated hypertensive patients [31]. Beat-to-beat BPV was associated with aortic stiffness and aortic pulsatility [32]. One study from China showed that within-visit DBP variability was associated with increased carotid IMT and internal carotid plaque in the normotensive population, and within-visit SBP variability was associated with internal carotid plaque in hypertensive patients undergoing antihypertensive therapy [33] (the maximum absolute difference between any two readings of three measurements was used to indicate within-visit BP variability). Furthermore, elevated daytime systolic BPV has been associated with an increased risk of developing early atherosclerosis [34] because the arterial walls of large vessels were more susceptible to intermittent stress than to continuous stress [35]. Beyond the relation to atherosclerosis, BPV variations within one visit were associated with a worse cardiovascular risk profile, including the prevalence of prediabetes and diabetes [36, 37]. In addition, carotid artery plaque is correlated with stroke [38]. Our study showed that a higher prevalence of carotid plaque in the WCE2.5 group (22.0% vs. 11.1% and 14.0%, *P*=0.073) may partly explain the relation, especially when the individuals in this group have carotid plaques simultaneously.

Arterial baroreflex sensitivity (BRS) is an important determinant of short-term regulation of blood pressure. BRS impairment leads to a higher BPV in a very short time [39]. The BRS is impaired after acute stroke [40], and impairment of the BRS can predict cardiovascular death in acute ischemic stroke patients in a long-term follow-up, independent of age, BP level, stroke severity, or stroke subtype [41]. The increase in BPV in hypertensive subjects may be partially explained by the diminished baroreflex function associated with increased stiffness and decreased compliance of large elastic arteries [42]. Therefore, it is questionable whether the increased BPV within a single visit is a cause or just an index of atherosclerosis. Hypertension, atherosclerosis, and aging could lead to arterial remodeling, which were often considered the predominant mechanisms responsible for a decreased BRS [43]. Thus, the WCE_2.5_ group and WCE were related to stroke possibly through an impaired BRS or accompanying impaired BRS, which needs further study to clarify.

The present study is not without its limitations. The subjects were volunteers, which may have led to selection bias. The small number of subjects in the extreme group WCE_2.5_ raises concerns regarding the robustness of the data, but the trend was also seen in the WCE_5_ group. The cause and effect relationship of this phenomenon to stroke are not definitive since our study was a cross-sectional study. Hence, long-term prospective studies are needed.

Our study showed that BPV within a single visit was correlated with stroke. We should recognize that BPV among the three measurements beyond some extent and direction might imply that the individual is at risk of stroke and that it is not just a phenomenon during blood pressure measurement. This proposed pattern should be further investigated as an easily obtainable BP biomarker with potential pathophysiologic and clinical relevance in stroke prevention.

## Figures and Tables

**Table 1:** Distribution of WCE.

	2.5th	5th	25th	50th	75th	95th	97.5th
WCE (mmHg)^a^	−4	−3	−1	1	2	4	6

^a^WCE refers to the first SBP measurement minus the mean of the second and the third measurements within a single visit.

**Table 2:** Comparison of the general characteristics of the three groups of WCE.

	WCE_2.5-97.5_ (*n* = 2874)	WCE_2.5_ (*n* = 41)	WCE_97.5_ (*n* = 57)	*p* ^*a*^
Age (years)	56 (49, 69)	56 (49, 68)	58 (46, 69)	0.995
Gender (male, *n*, %)	1302 (45.3)	18 (43.9)	28 (49.1)	0.833
BMI (kg/m^2^)	25.2 (23.1, 27.5)	24.5 (21.5, 27.9)	25.4 (22.5, 27.5)	0.547
SBP (mmHg)	131 (119, 144)	130 (115, 148)	136 (126, 150)	0.035
DBP (mmHg)	79 (71, 85)	81 (71, 87)	79 (70, 87)	0.974
HR (bpm)	76 (70, 84)	76 (67, 84)	76 (72, 81)	0.957
cIMT (mm)	0.63 (0.49, 0.75)	0.61 (0.45, 0.69)	0.61 (0.46, 0.72)	0.221
Ba-PWV (cm/s)	1602 (1360, 1892)	1814 (1393, 2040)	1702 (1430, 2037)	0.057
TC (mmol/l)	5.10 (4.50, 5.78)	5.09 (4.38, 5.83)	5.30 (4.77, 5.90)	0.348
TG (mmol/l)	1.31 (0.96, 1.92)	1.53 (1.06, 2.23)	1.41 (1.02, 1.94)	0.272
Smoking (*n*, %)	722 (25.1)	13 (31.7)	19 (33.3)	0.238
Hypertension (*n*, %)	1368 (47.6)	24 (58.5)	33 (57.9)	0.120
Antihypertensive drug usage (*n*, %)	830 (29.0)	14 (34.1)	17 (29.8)	0.632
CKD (*n*, %)	141 (4.9)	1 (2.4)	1 (1.8)	0.420
MI (*n*, %)	110 (3.8)	3 (7.3)	2 (3.5)	0.511
Diabetes (*n*, %)	318 (11.1)	9 (22.0)	8 (14.0)	0.073
Stroke (*n*, %)	198 (6.9)	7 (17.1)	3 (5.3)	0.035
Carotid plaque (*n*, %)	1477 (51.6)	22 (53.7)	19 (33.3)	0.023

^a^ *P* for comparison among the three WCE groups.

**Table 3:** Univariate logistic regression of predictors for stroke.

	OR (95% CI)	*P*
Age	1.08 (1.07, 1.10)	<0.001
Gender^a^	0.71 (0.53, 0.94)	0.017
Smoking	1.06 (0.77, 1.46)	0.713
Hypertension	8.57 (5.64, 13.01)	<0.001
Diabetes	2.92 (2.09, 4.09)	<0.001
BMI	1.06 (1.02, 1.10)	0.004
TC (per mmol/l increasing)	0.99 (0.89, 1.11)	0.890
TG (per mmol/l increasing)	1.05 (0.92, 1.19)	0.469
SBP (per mmHg increasing)	1.03 (1.02, 1.03)	<0.001
DBP (per mmHg increasing)	1.01 (1.00, 1.02)	0.253
cIMT thickening	3.15 (1.83, 5.44)	<0.001
Carotid plaque	3.15 (2.27, 4.37)	<0.001
ba-PWV abnormality	8.95 (4.38, 18.29)	<0.001
WCE (per mmHg increasing)	0.94 (0.89, 0.99)	0.032
WCE_5_^b^	1.71 (0.94, 3.11)	0.077
WCE_95_^b^	0.68 (0.31, 1.48)	0.330
WCE_2.5_^c^	2.78 (1.22, 6.36)	0.015
WCE_97.5_^c^	0.75 (0.23, 2.42)	0.632

^a^Male as the reference. ^b^Compared with the group of WCE_5-95_. WCE_5-95_ refers to 5–95th percentiles of WCE; WCE_5_ refers to 0–4.9th percentiles of WCE; WCE_95_ refers to 95.1–100th percentiles of WCE. ^c^Compared with the group of WCE_2.5-97.5._ WCE_2.5-97.5_ refers to 2.5–97.5th percentiles of WCE; WCE_2.5_ refers to 0–2.4th percentiles of WCE; WCE_97.5_ refers to 97.6–100th percentiles of WCE.

**Table 4:** Multiple logistic regression of the relation between WCE and stroke incidence.

	OR (95% CI)^a^	*P*
WCE	0.93 (0.87, 0.99)	0.036
WCE_2.5_	3.12 (1.22, 7.96)	0.017
WCE_97.5_	0.72 (0.16, 3.916)	0.717

^a^Adjusted for age, BMI, gender, SBP, diabetes, hypertension, TC, antihypertensive drug usage, smoking status, cIMT  thickening, carotid plaque, ba-PWV abnormality, CKD, and MI.

## Data Availability

The data used to support the findings of this study are available from the corresponding author upon request.
